# Assessment of an organ‐based tube current modulation in thoracic computed tomography

**DOI:** 10.1120/jacmp.v13i2.3731

**Published:** 2012-03-08

**Authors:** Kosuke Matsubara, Mai Sugai, Asami Toyoda, Haruka Koshida, Keita Sakuta, Tadanori Takata, Kichiro Koshida, Hiroji Iida, Osamu Matsui

**Affiliations:** ^1^ Department of Quantum Medical Technology, Faculty of Health Sciences Kanazawa University Kanazawa Japan; ^2^ Department of Radiological Technology, School of Health Sciences Kanazawa University Kanazawa Japan; ^3^ Department of Radiology, Faculty of Medicine Kanazawa University Kanazawa Japan; ^4^ Department of Radiological Technology Kanazawa University Hospital Kanazawa Japan

**Keywords:** computed tomography, breast, radiation dosage, tube current modulation

## Abstract

Recently, specific computed tomography (CT) scanners have been equipped with organ‐based tube current modulation (TCM) technology. It is possible that organ‐based TCM will replace the conventional dose‐reduction technique of reducing the effective milliampere‐second. The aim of this study was to determine if organ‐based TCM could reduce radiation exposure to the breasts without compromising the image uniformity and beam hardening effect in thoracic CT examinations. Breast and skin radiation doses and the absorbed radiation dose distribution within a single section were measured with an anthropomorphic phantom and radiophotoluminescent glass dosimeters using four approaches to thoracic CT (reference, organ‐based TCM, copper shielding, and the combination of the above two techniques, hereafter referred to as the combination technique). The CT value and noise level were measured using the same calibration phantom. Organ‐based TCM and copper shielding reduced radiation doses to the breast by 23.7% and 21.8%, respectively. However, the CT value increased, especially in the anterior region, using copper shielding. In contrast, the CT value and noise level barely increased using organ‐based TCM. The combination technique reduced the radiation dose to the breast by 38.2%, but greatly increased the absorbed radiation dose from the central to the posterior regions. Moreover, the CT value increased in the anterior region and the noise level increased by more than 10% in the entire region. Therefore, organ‐based TCM can reduce radiation doses to breasts with only small increases in noise levels, making it preferable for specific groups of patients, such as children and young women.

PACS numbers: 87.53.Bn; 87.57.Q‐; 87.57.qp

## I. INTRODUCTION

Computed tomography (CT) is a medical imaging technique that is very effective for detecting lung cancer at an early stage.^(^ ^1^ ^,^ ^2^ ^)^ However, the increase in CT examination frequency and higher doses of radiation in CT examinations compared with other X‐ray diagnostic procedures have raised concerns about radiation exposure to patients.^(3)^ According to the International Commission on Radiological Protection Publication 103, the tissue weighting factor for the breasts has been raised from 0.05 to 0.12,^(4)^ implying that overexposure to the breasts should be avoided.

To reduce radiation doses to the breasts, dose‐reduction techniques, such as selective breast shields that are designed to cover both breasts, have been used. Hopper et al.^(5)^ and Fricke et al.^(6)^ recommended bismuth shielding to achieve better radiation protection for patients undergoing thoracic CT examinations. However, Vollmar et al.^(7)^ reported that bismuth shielding compromised image quality, increased noise levels, and introduced streak artifacts; therefore, the use of bismuth shielding was not recommended. In addition, Takada et al.^(8)^ reported that shields made from zinc, copper, and iron were more effective for dose reduction than a shield made from bismuth because the former have lower atomic numbers and lower yields of characteristic X‐rays.

Tube current modulation (TCM) techniques adjust the tube current along the angular (xy‐axis), longitudinal (z‐axis), or both directions, to optimize radiation dose to patients.^(^ ^9^ ^,^ ^10^ ^)^ Recently, specific CT scanners have been equipped with an organ‐based TCM feature. The technique involves reduction of the X‐ray tube output in real time when it is directly in front of breasts or other dose‐sensitive organs, such as thyroid gland and eye lens. When it is used in combination with xy‐axis and z‐axis TCMs, the organ‐based and z‐axis TCMs function, while the xy‐axis TCM does not function. We believe that the organ‐based TCM technique may replace breast shielding as the preferred dose‐reduction technique to breasts.

In this study, we compared the dose, CT value, and noise level of three dose‐reduction techniques to breasts in thoracic CT examinations. The techniques were organ‐based TCM, breast shielding using copper, and the combination of the above two techniques, hereafter referred to as the combination technique. Our goal in this study was to determine if organ‐based TCM could reduce radiation doses to breasts without compromising the image uniformity and beam hardening effect in thoracic CT examinations.

## II. MATERIALS AND METHODS

### A. CT system and phantom

A 128‐detector dual‐source CT SOMATOM Definition Flash (Siemens Healthcare, Erlangen, Germany) was used. The effective energy at a tube voltage of 120 kV was 55.7 keV (half‐value layer of 8.23 mm aluminum) measured using the aluminum absorption method.^(11)^

**Figure 1:**
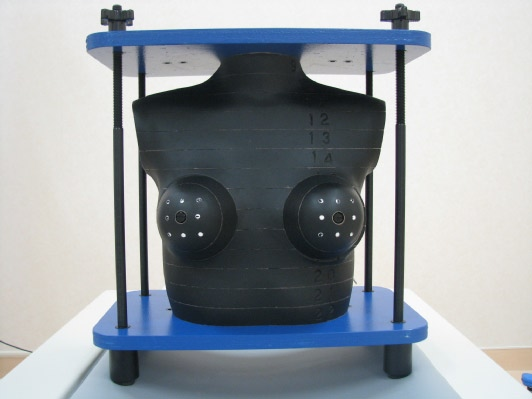
The anthropomorphic female thoracic phantom used in our study. The entire phantom was cut into thin transverse sections having grids of holes for placement of small dosimeters.

For radiation dose evaluation, the RAN110 (The Phantom Laboratory, Salem, NY, USA) anthropomorphic female thoracic phantom onto which two breast sections were mounted was used (Fig. 1). The assembly had an embedded natural skeleton, an epoxy resin‐based lung substitute, and an isocyanate rubber‐based muscle substitute. To evaluate variations of the CT values and noise levels, a Catphan‐600 calibration phantom (The Phantom Laboratory) was used. The phantom consisted of five modules, and the image uniformity module (CTP486: The Phantom Laboratory) was used in this study.

### B. Dosimeters and dose calibration

Radiophotoluminescent glass dosimeters (RPLDs) (GD‐302M; Chiyoda Technol, Tokyo, Japan) were used to estimate the radiation dose absorbed by the phantom. The RPLDs were 1.5 mm in diameter and 12 mm in length. According to the basic characteristic data of the RPLDs provided by the manufacturer, the variation in sensitivity among the RPLDs was approximately ≤2% with ≥1mGy of C137s gamma irradiation, and the reproducibility of RPLD measurements was ≤1% with ≥0.1mGy when the same RPLD was read out 10 times at the same readout position.

Dose calibration was carried out against an ionizing dosimeter (Ramtec 1500B; Toyo Medic, Tokyo, Japan) with a $3\;{\text{cm}}^{3}$ ion chamber attached to a 120 kVp (effective energy 55.0 keV; half‐value layer of 8.08 mm aluminum) diagnostic X‐ray beam. The chamber and RPLDs were placed side by side at the same distance from the X‐ray focus in an irradiated field. The ionizing dosimeter had previously been calibrated at a laboratory accredited by the Japan Quality Assurance Organization. The RPLDs were annealed at 400°C for 30 min prior to each exposure. After each exposure, the RPLDs were further heated to 70°C for 30 min and read using a FGD‐1000 reader (Chiyoda Technol) in accordance with the manufacturer's recommended protocol.

### C. Measurement of breast and skin radiation doses

**Figure 2:**
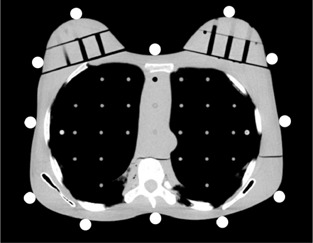
The arrangement of the radiophotoluminescent glass dosimeters (RPLDs) for measurement of the skin radiation dose. They were placed every 30° around the section, which included locations corresponding to the nipples.

After obtaining localizer radiographs, the positions of the four corners of the phantom were marked on a CT table using tape and a felt‐tip pen to maintain the phantom in the same position for subsequent thoracic CT acquisitions; the phantom was then taken off the bed. Twelve RPLDs were placed at locations corresponding to the breasts and another 12 at locations corresponding to the skin (Fig. 2). The phantom was then replaced on the bed using the previously made reference marks. These procedures were performed to exclude the doses of the localizer radiographs from the results. Two RPLDs were used to measure the background radiation.

**Figure 3:**
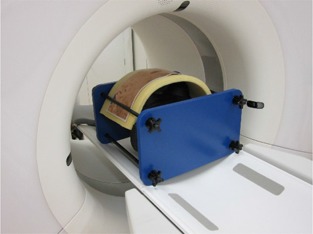
Image showing the copper shielding method for measurement of the breast and skin radiation doses. The shield was positioned over foam pads that were placed on the phantom.

A reference thoracic CT scan was performed with standard values used in our institution as follows: 120 kV with fixed effective mAs of 140 (without using TCM), 3.0 mm slice thickness, 0.5 sec rotation time, 0.6 pitch factor, 128×0.6mm slice collimation, 9.45 mGy displayed volume CT dose index, and a B36f‐reconstruction kernel (HeartView medium with advanced smoothing algorithm; Siemens Healthcare). In addition, thoracic CT with organ‐based TCM (X‐CARE; Siemens Healthcare), copper shielding (using a 0.2 mm thick copper plate, 15 cm height, 50 cm width, and 99.9% purity), and the combination technique were also performed using the same values as used for the reference. The copper shield was used by placing it over 2 cm thick foam pads to lift it away from the phantom in an attempt to minimize radiation scatter to the phantom and decrease the potential for artifacts (Fig. 3). To achieve more accurate RPLD readings, each measurement was performed in duplicate using two sets of RPLDs.

Breast and skin radiation doses were calculated by multiplying the calibrated dose values that were obtained from the reader by the mass energy coefficient ratio of each tissue to air.^(12)^

### D. Measurement of the absorbed radiation dose distribution within a single section

**Figure 4:**
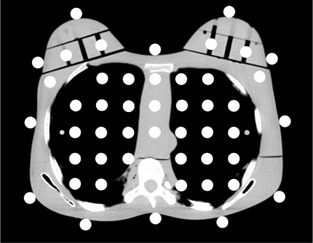
The arrangement of the RPLDs for measurement of the absorbed radiation dose distribution within the section including locations corresponding to the nipples.

The phantom was taken off the bed after obtaining localizer radiographs, 36 RPLDs were placed within all of the holes of one section, and 12 were pasted around the section (Fig. 4). The phantom was then replaced on the bed, and measurement of the absorbed radiation dose distribution within a single section was performed using separate sets of dosimeters when the thoracic CTs were performed using the four approaches (the reference, organ‐based TCM, copper shielding, and the combination technique) with the same parameters as those used for measurement of the breast and skin radiation doses.

The absorbed radiation doses within the phantom were calculated by multiplying the calibrated dose values by the mass energy coefficient ratio of soft tissue to air; the surface‐absorbed doses were calculated by the mass energy coefficient ratio of skin to air.^(^ ^12^ ^,^ ^13^ ^)^ A graph of each absorbed radiation dose distribution was drawn using graphing software (Origin 8.1; OriginLab, Northampton, MA). The software defined the contours of the regions based on dose measurements at discrete positions using a Delaunay triangulation algorithm.^(14)^

### E. Measurement of the CT values and noise levels

**Figure 5:**
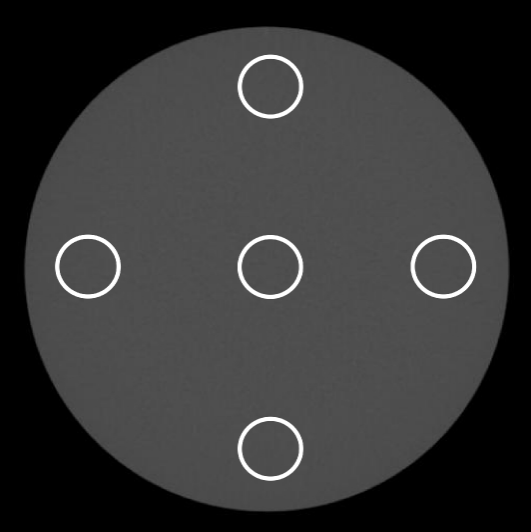
The circular regions of interest (2.0 cm diameter) for measurement of the computed tomography (CT) value and noise level.

Thoracic CTs were performed using the four approaches with the same parameters as those used for measurement of the breast and skin radiation doses. Thereafter, the CT values and noise levels were measured in five regions of interest (Fig. 5) in the central ten slices of the scanned volume of the phantom module. The noise level was recorded as the standard deviation (SD) in Hounsfield units. These results were presented as the average values over every slice. Advantage Workstation version 4.4 software (GE Healthcare, Milwaukee, WI) was used to measure the CT and SD values.

## III. RESULTS

### A. Radiation dose

The mean radiation doses to the breasts over 12 measured points using the four approaches to thoracic CT are shown in Table 1. These results indicated that the organ‐based TCM and copper shielding approaches reduced the radiation dose to the breasts almost equally and that the combination technique was the most effective.

**Table 1:** Radiation dose to the breast using the four approaches to thoracic CT.

	*Radiation Dose to the Breast (mGy)*		
*Approach*	*Mean*	*SD*	*Diff. to Ref. (%)*
Reference	9.59	0.77	
Organ‐based tube current modulation	7.32	0.78	−21.8
Copper shielding	7.50	0.91	−23.7
Combination technique	5.92	0.85	−38.2

**Figure 6:**
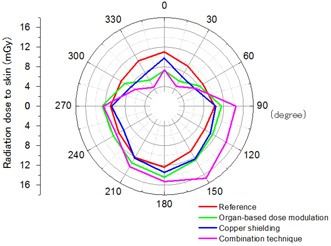
The results for the skin radiation dose at each point measured using the four approaches to thoracic CT.

The mean radiation dose to the skin at each measured point using the four approaches to thoracic CT are shown in Fig. 6. Compared to the reference, the three dose‐reduction techniques to the breast reduced the frontal (from 300° to 60°) radiation dose to the skin; however, the organ‐based TCM and combination techniques increased the lateral and dorsal (from 90° to 270°) radiation doses to the skin.

**Figure 7:**
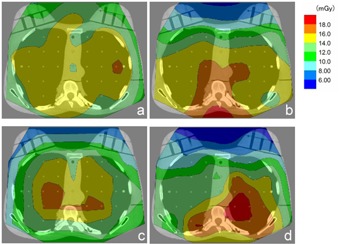
The results of the absorbed radiation dose distributions within a single section using the four approaches to thoracic CT: (a) reference, (b) organ‐based TCM, (c) copper shielding, and (d) combination technique.

The absorbed radiation dose distributions within single sections using the four CT approaches are shown in Fig. 7. The mean absorbed radiation doses within a single section over 48 measured points were 13.7, 13.8, 12.5, and 12.5 in the reference, the organ‐based TCM, the copper shielding, and combination technique, respectively. Compared with the reference, the organ‐based TCM reduced the absorbed radiation dose in the anterior region and increased it in the posterior region. Copper shielding also decreased the absorbed radiation dose in the anterior region and increased it in the central region. The combination technique was the most effective approach for reducing the absorbed radiation dose in the anterior region. Among the four approaches used, the combination technique gave the highest absorbed radiation dose of thoracic CT from the central to the posterior regions.

### B. CT values and noise level

The CT values at five points measured using the four approaches to thoracic CT are shown in Table 2. Compared with the reference, the copper shielding and combination techniques increased the CT values, especially in the 12 o'clock position, but organ‐based TCM changed them only slightly.

**Table 2:** CT values using the four approaches to thoracic CT.

	*Center*		*12 O'clock*		*Position 3 O'clock*		*6 O'clock*		*9 O'clock*	
*Approach*	*CT value (HU)*	*Diff. to ref. (HU)*	*CT value (HU)*	*Diff. to ref. (HU)*	*CT value (HU)*	*Diff. to ref. (HU)*	*CT value (HU)*	*Diff. to ref. (HU)*	*CT value (HU)*	*Diff. to ref. (HU)*
Reference	11.4		4.1		4.5		4.9		4.6	
Organ‐based tube current modulation	11.0	−0.4	4.0	−0.1	4.2	−0.3	4.9	0.0	4.0	−0.6
Copper shielding	15.5	4.1	15.1	11.0	8.8	4.4	5.8	0.9	9.7	5.1
Combination technique	15.1	3.7	14.7	10.6	8.5	4.0	5.8	0.9	9.5	4.9

The SD values at the five points measured using the four approaches to thoracic CT are shown in Table 3. Compared with the reference, the SD values increased from −0.8% to 5.4% using organ‐based TCM, which was smaller than the increases resulting from the other approaches (from 4.5% to 13.8% using copper shielding and from 10.5% to 15.5% using the combination technique).

**Table 3:** Noise property using the four approaches to thoracic CT.

	*Center*		*12 O'clock*		*Position 3 O'clock*		*6 O'clock*		*9 O'clock*	
*Approach*	*SD value (HU)*	*Diff. to ref. (%)*	*SD value (HU)*	*Diff. to ref. (%)*	*SD value (HU)*	*Diff. to ref. (%)*	*SD value (HU)*	*Diff. to ref. (%)*	*SD value (HU)*	*Diff. to ref. (%)*
Reference	7.1		5.8		5.8		5.9		5.9	
Organ‐based tube current modulation	7.4	4.4	6.1	4.8	5.8	−0.6	6.2	5.4	5.8	−0.8
Copper shielding	7.7	8.7	6.1	4.5	6.6	13.8	6.6	12.2	6.6	12.6
Combination technique	7.9	10.6	6.5	11.4	6.6	12.9	6.8	15.5	6.5	10.5

## IV. DISCUSSION

The aim of this study was to determine if organ‐based TCM could reduce radiation doses to breasts without compromising the image uniformity and beam hardening effect in thoracic CT examinations. Our results indicated that organ‐based TCM and copper shielding reduced radiation doses to the breasts almost equally; however, the CT value increased, especially in the anterior region, using the copper shielding approach. In contrast, the values using organ‐based TCM changed only slightly.

Fundamentally, shielding has very different effects on radiation exposures to patients for frontal projection compared with dorsal projection.^(15)^ Copper shielding results in attenuation or scatter of the incoming (anteroposterior projection) and outgoing (posteroanterior projection) X‐ray beam and emission of characteristic X‐rays. Our study indicated that copper shielding of the incoming X‐rays helped to reduce the radiation dose to the breasts; however, the disadvantages were that it also increased the CT value and noise level. It is not apparent that these would be significant effects from a clinical perspective, but there is a possibility that these disadvantages may obstruct diagnoses of breast or mediastinal diseases. Moreover, we assumed that the scatter and characteristic X‐rays using the copper shielding approach were the main reasons for the increased skin radiation dose from 90° to 270° shown in Fig. 6 and the absorbed radiation dose in the central region shown in Fig. 7. However, the mean absorbed radiation dose within a single section was lower using copper shielding than in the reference. Therefore, we think copper shielding in thoracic CT is useful only for patients who probably do not have breast or mediastinal diseases (e.g., patients who receive CT screenings for lung cancer). Because we did not prove why the scatter and characteristic X‐rays increased the absorbed radiation dose in the central region without increasing it near the copper shielding area (e.g., the breast), this anomaly should be investigated in the near future.

When organ‐based TCM is applied, the output of the X‐ray tube is reduced when it reaches the frontal position to reduce the breast radiation dose and increased when it reaches the dorsal position to keep the noise level low. Our results showed that the CT values and noise levels barely increased compared with the reference, and the mean absorbed radiation dose within a single section using organ‐based TCM was almost the same as the reference. We are certain that this overall dose modulation increased the skin radiation dose from 90° to 270° shown in Fig. 6 and the absorbed radiation dose in the posterior region shown in Fig. 7. Doody et al.^(16)^ suggested that exposure to multiple diagnostic radiographic examinations during childhood and adolescence would increase the risk of breast cancer among women with scoliosis. Therefore, organ‐based TCM is preferable for specific groups of patients, such as children and young women, in whom the risk of breast cancer might be increased by thoracic CT.

By combining organ‐based TCM and shielding techniques, more scatter and characteristic X‐rays should be generated because of the increased output of the X‐ray tube in the dorsal position. We believe that this was the cause of the greatly increased absorbed radiation dose observed from the central to the posterior regions. We could not demonstrate the reason for the uneven distribution of the skin radiation dose from 90° to 270° shown in Fig. 6 and the absorbed radiation dose in the posterior region shown in Fig. 7, but we assumed that it was the combination of increased scatter and characteristic X‐rays in addition to the spiral scan. Moreover, the increases in the CT value in the anterior region and noise level of more than 10% over the entire region cannot be disregarded. There is a possibility that these disadvantages could obstruct diagnoses of various thoracic diseases; therefore, we think the combination technique is not a practical approach.

There were several limitations to our study. First, only one specific anthropomorphic phantom and one specific calibration phantom were used. Results can differ according to the size, shape, and composition of each phantom. An evaluation of image quality using an elliptical body sized uniform phantom may be needed because using a perfectly circular calibration phantom to score the image quality may be biased. In addition, the breast parts of an anthropomorphic phantom are not malleable, but human breasts are malleable and are pulled posterior and lateral. Judging from the dose distribution in Fig. 6, the organ‐based TCM might actually increase the breast dose if the breasts are pulled posterior and lateral. Second, although a limited number of RPLDs was evenly distributed at locations that corresponded to the breast and the skin, the actual breast and skin radiation doses could have been different. Third, only a copper shield of 0.2 mm thickness was used in our study, according to the methods of Takata et al.^(8)^ The dose reduction rate can change depending on the thickness of the shields. In addition, breast shielding is generally achieved by applying latex sheets that contain bismuth.^(^ ^5^ ^–^ ^7^ ^,^ ^15^ ^)^ Therefore, to achieve more specific results, it would be necessary to perform similar examinations using copper shields of different thicknesses and shields made from other metals. Fourth, although there are many methods for measurement of image quality in CT, we measured only the CT value and noise level for evaluation of the image uniformity and beam hardening effect. Finally, although xy‐axis and z‐axis TCMs are useful for reducing the radiation dose to the breast in thoracic CTs,^(^ ^7^ ^,^ ^17^ ^,^ ^18^ ^)^ it was not used in combination with the four approaches to thoracic CT in the present study. However, the use of breast shields in conjunction with xy‐axis and z‐axis TCMs results in an increase in tube current to offset the effects of the shield. This is because the automatic exposure control system installed in the CT instrument used in our study performs continual monitoring of patient attenuation and adapts the tube current settings in real time during the scan.^(^ ^15^ ^,^ ^19^ ^)^

## V. CONCLUSIONS

Organ‐based TCM can reduce radiation doses to breasts and involves only a small change in the noise level. The application only of organ‐based TCM is recommended for specific groups of patients, such as children and young women, who might face an increased risk of breast cancer by undergoing repeated thoracic CTs.

## ACKNOWLEDGMENTS

We thank Hisashi Nishibayashi, Katharina Otani, Tomoko Fujihara, and Takeyoshi Nozawa of Siemens Japan K.K. for their kind support. We also thank our colleagues Sho Wakumura, Masato Saga, Hiroshi Fujikawa, Haruki Hosoki, and Ayano Yuasa in the Koshida Laboratory of Kanazawa University for fruitful discussions.
